# Comparative Assessment of the Long-Term Efficacy of Home-Based Versus Center-Based Cardiac Rehabilitation

**DOI:** 10.7759/cureus.23485

**Published:** 2022-03-25

**Authors:** Nso Nso, Mahmoud Nassar, Yolanda Mbome, Kelechi E Emmanuel, Anthony Lyonga Ngonge, Solomon Badejoko, Shahzad Akbar, Ian Landry, Mostafa Alfishawy, Most Munira, Vincent Rizzo

**Affiliations:** 1 Internal Medicine, Icahn School of Medicine at Mount Sinai, Queens Hospital Center, New York, USA; 2 Internal Medicine, Icahn School of Medicine at Mount Sinai, New York City Health and Hospitals/Queens, New York, USA; 3 Internal Medicine, Richmond University Medical Center, New York, USA; 4 Internal Medicine, University of Pittsburgh Medical Center Pinnacle, Harrisburg, USA; 5 Department of Medicine, State University of New York Upstate Medical University, New York, USA; 6 Internal Medicine, St. Joseph’s Medical Center, Stockton, USA; 7 Internal Medicine, Kettering Medical Center, Dayton, USA; 8 Medicine, Icahn School of Medicine at Mount Sinai, New York City Health and Hospitals/Queens, New York, USA; 9 Infectious Diseases, Infectious Diseases Consultants and Academic Researchers of Egypt (IDCARE), Cairo, EGY; 10 Cardilogy/Medicine, Weill Cornell Medicine, New York, USA; 11 Cardiology, Queens Hospital Center, New York, USA; 12 Internal Medicine, Icahn School of Medicine at Mount Sinai, Queens Hospital Center, New York City, USA

**Keywords:** cardiac, center-based cardiac rehab, home-based cardiac rehab, cardiovascular, cardiac rehabilitation

## Abstract

Cardiac rehabilitation programs support the health, wellness, and recovery of patients with cardiovascular conditions. This systematic review attempts to expand these findings while analyzing the latest randomized controlled trials (RCTs) focusing on the long-term advantages of home/center-based cardiac rehabilitation interventions. This study also comparatively analyzes the benefits of opting for home-based cardiac rehabilitation instead of center-based measures to improve the long-term clinical outcomes of cardiac patients. We extracted and analyzed 10 studies (based on 1,549 cardiac patients) concerning the therapeutic efficacy of center/home-based cardiac rehabilitation interventions. The included studies complied with the year range of 2000-2021. The risk of bias assessment was undertaken using the Cochrane Risk-of-Bias tool to evaluate random sequence generation, allocation concealment, blinding of subjects, outcome data completeness, and selective reporting patterns concerning the included RCTs.

The findings of our systematic review confirmed the capacity of a home-based cardiac rehabilitation program to effectively improve left ventricular ejection fraction, health-related quality of life, physical fitness, recovery rate, self-efficacy, sedentary lifestyle, physical activity, satisfaction level, functional capacity, social support, and hemodynamic parameters of patients with cardiovascular diseases. Home-based cardiac rehabilitation had the potential to minimize the levels of triglycerides, anxiety, depression, waist circumference, and body mass index/weight of cardiac patients.

The results of our systematic review affirmed the long-term therapeutic efficacy of a home-based cardiac rehabilitation program compared to a center-based cardiac rehabilitation program for adult cardiac patients.

## Introduction and background

Cardiac rehabilitation programs rely on focused approaches to minimize the physiological and psychological stresses that impact patients’ quality of life and mortality risk with cardiovascular complications [1]. Cardiac rehabilitation interventions based on exercise training, health behavior modification procedures, and patient education measures help improve the clinical outcomes in the setting of heart disease [2]. Center-based cardiac rehabilitation provides an opportunity for cardiac patients to attend interactive health support sessions to minimize their health risks and enhance their treatment outcomes. They also reduce the risk of hospital readmissions and cardiovascular mortality for patients with post-acute myocardial infarction, coronary artery bypass graft status, and other cardiac morbidities. Center-based cardiac rehabilitation programs in hospital settings, gymnasiums, clinics, healthcare centers, and sports complexes help improve cardiac patients’ quality-adjusted life years [3]. Home-based cardiac rehabilitation programs need administration inside the residential locations of cardiac patients and increase their accessibility to health support interventions [4]. Technology-assisted, home-based cardiovascular rehabilitation approaches further help personalize patient support interventions. They provide the advantage of extending healthcare support to remotely located cardiac patients. In extending home-based cardiac rehabilitation programs, telemedicine interventions expand their administration across larger patient populations [5]. Clinical studies have advocated similar effectiveness of center-based and home-based cardiac rehabilitation measures in improving cardiac patients’ lifestyle and wellness perceptions with revascularization status and clinical history of acute myocardial infarction [6]. A recent meta-analysis/systematic review by Imran et al. (2019) advocated the short-term efficacy of home/center-based hybrid cardiac rehabilitation programs in improving heart failure patients’ lifestyle and functional capacity [7].

Similarly, a systematic review by Anderson et al. (2017) affirmed the comparable efficacy of home-based versus center-based cardiac rehabilitation programs in elevating the lifestyle and clinical outcomes of patients with a clinical history of heart failure, revascularization, and myocardial infarction [8]. These systematic reviews/meta-analyses emphasize the short-term potential of center/home-based cardiac rehabilitation programs in improving the wellbeing outcomes of cardiac patients. However, these studies do not confirm the superiority of home-based cardiac rehabilitation approaches over facility-based cardiac rehabilitation interventions. Our systematic review aims to expand the outcomes of these studies and understand the long-term efficacy of home-based versus center-based cardiac rehabilitation interventions for cardiac patients.

Methodology

We utilized the Preferred Reporting Items for Systematic Reviews and Meta‐Analyses (PRISMA) framework to perform our systematic review [9].

Eligibility Parameters

We included randomized controlled trials (RCTs) targeting the therapeutic efficacy of home-based versus center-based cardiac rehabilitation interventions for adults over 18 years of age. The assessment of the long-term efficacy of the included cardiac rehabilitation approaches relied on analyzing the following end-points/outcome measures: left ventricular ejection fraction (LVEF), health-related quality of life (HRQOL), physical fitness, recovery rate, self-efficacy, sedentary lifestyle, moderate-to-vigorous-intensity physical activity, patient satisfaction level/sense of wellness, reduction in triglycerides, decrease in anxiety levels, reduction in depression, decrease in waist circumference, reduction in body mass index/weight, reduction in social isolation/enhancement in social support, enhancement of hemodynamic parameters, and enhancement in functional capacity.

Electronic Searches

The exploration of the articles of interest was independently undertaken by two authors across Psych-Info, Google Scholar, PubMed/Medline, Embase, CINAHL, and Cochrane Library. Relevant RCTs published between 2000 and 2021 were considered for inclusion in our systematic review. The research process to retrieve the articles of interest was undertaken on May 5th and 6th, 2021. The Medical Subject Headings (MeSH) terms included “home-based cardiac rehabilitation,” “center-based cardiac rehabilitation,” “facility-based cardiac rehabilitation,” “cardiac patients,” “cardiac rehabilitation,” “exercise,” “fitness training,” “exertion,” “heart failure,” “exercise therapy,” and “telemonitoring.” We utilized Boolean operators to run various search combinations of the MeSH terms for obtaining the targeted articles. We confined our article search to the English language and extracted full-text articles satisfying the eligibility criteria.

Data Collection and Assessment

Two dedicated authors collected the data based on the included outcome variables. One author analyzed the outcomes of the included studies concerning the selected cardiac rehabilitation approaches and their therapeutic potential for patients with cardiovascular conditions. We excluded studies devoid of cardiac rehabilitation interventions, systematic reviews, meta-analyses, case studies, opinion papers, scoping reviews, and cohort studies. The authors mutually resolved their differences in opinions concerning data collection and developed an agreed-upon consensus for the systematic review of the included RCTs.

Two independent authors collected the inferences, outcomes, and other attributes of the selected RCTs on an optimized data collection form. The authors comprehensively analyzed the details of the cardiac rehabilitation interventions, including their center/home-based status and types of support measures/activities. The authors recorded their findings on a digital diary and transferred the data and outcomes to the study table.

Risk of Bias Assessment

**Figure 1:**
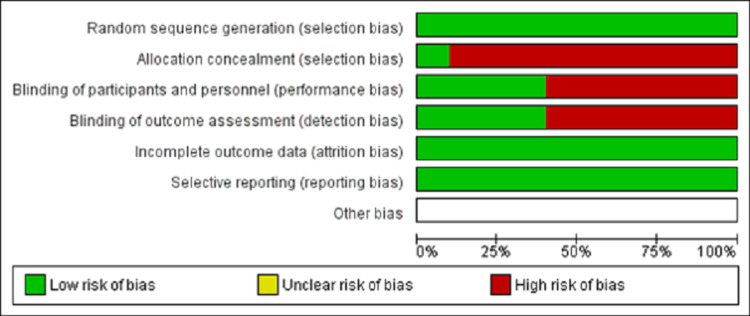
Risk of bias assessment of the included studies.

**Figure 2:**
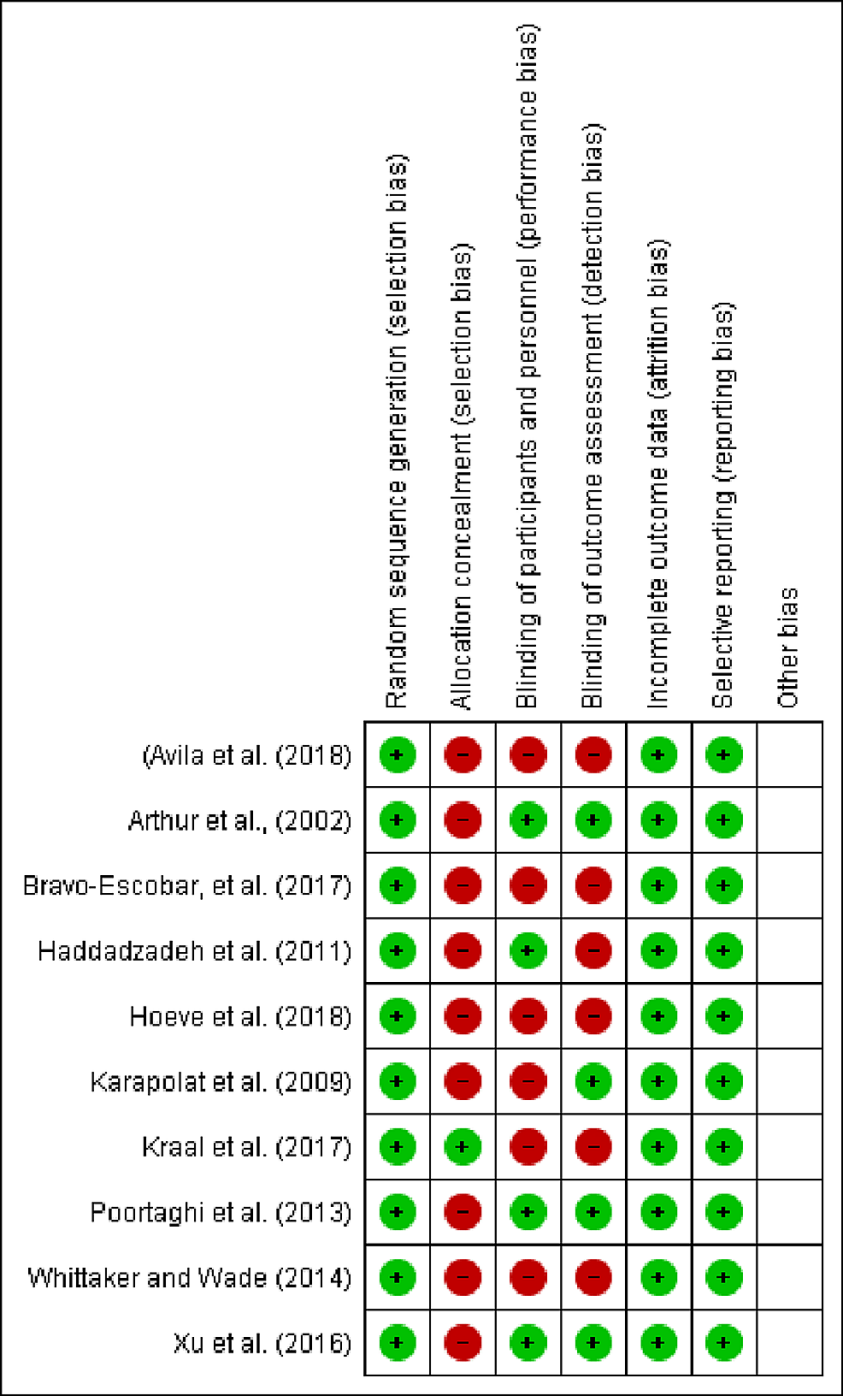
Risk of bias assessment of the included studies.

Two authors independently utilized the Cochrane Risk-of-Bias tool to investigate random sequence generation effectively, allocation concealment, blinding of subjects, outcome data completeness, and selective reporting patterns concerning the included RCTs (Figures 1, 2) [10]. They also evaluated equity in the administration of center/home-based cardiac rehabilitation interventions.

Outcome Measurement

Two authors categorically analyzed differences between home-based and center-based cardiac rehabilitation programs in the context of preselected variables. They considered sample sizes, mean differences, and continuous variables to examine the outcome data.

Missing Data

The authors collaborated to exclude abstract-only articles or studies with incomplete or missing information. The studies lacking the selected outcome variables were also summarily excluded from our systematic review.

## Review

**Figure 3:**
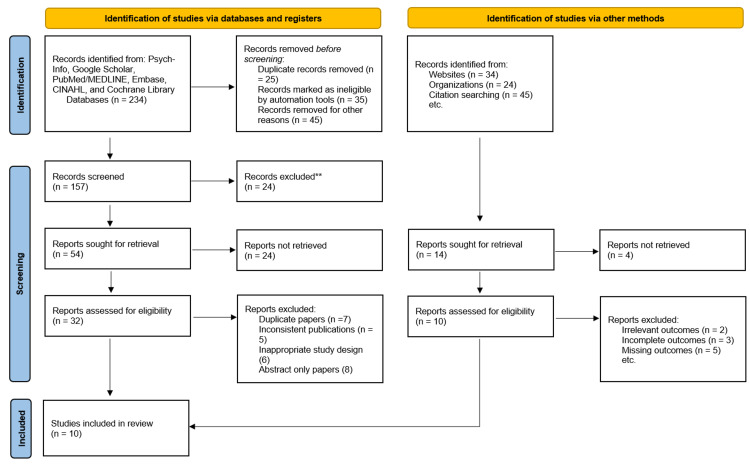
PRISMA flow diagram of the study screening process. PRISMA: Preferred Reporting Items for Systematic Reviews and Meta‐Analyses

We extracted and analyzed 10 studies (based on 1,549 cardiac patients) concerning the therapeutic efficacy of center/home-based cardiac rehabilitation measures (Figure 3). The cardiac rehabilitation programs investigated by the included studies included the following interventions [1]: exercise training, physical activity counseling, psychosocial management, tobacco cessation measures, diabetes management, lipid management, blood pressure management, weight/body mass index management, and nutritional counseling.

Three studies revealed the therapeutic efficacy of home-based exercise training intervention for enhancing the myocardial contractility of cardiac patients [11,15,19]. Their findings affirmed the comparable efficacy of home-based and center-based rehabilitation measures in elevating LVEF in patients with coronary artery disease status. Two studies confirmed the potential of home-based and telemonitoring-oriented, home-based programs to improve patients’ moderate-to-vigorous-intensity physical activity levels with cardiovascular complications [12,17]. These studies affirmed the wellness benefits of home-based interventions over center-based measures for cardiac patients. Four studies affirmed the potential of home-based cardiac rehabilitation measures in improving patients’ HRQOL with ischemic heart disease, coronary artery disease, heart failure, myocardial infarction, angina, and coronary artery bypass graft status [12,17,19,20]. One study negated the efficacy of telephone-based cardiac rehabilitation compared to center-based cardiac rehabilitation in elevating the sedentary lifestyle of patients with acute coronary syndrome [16].

Two studies affirmed the effectiveness of home/telemonitoring-based cardiac rehabilitation in improving the overall physical fitness of cardiac patients in the longer term [12,17]. One study advocated the benefit of telemonitoring-oriented cardiac rehabilitation measures to improve the long-term satisfaction level of cardiac patients [17]. Two studies indicated the depression management capacity of home-based cardiac rehabilitation measures compared to the center-based cardiac rehabilitation interventions [18,19]. One study confirmed the role of telemedicine-based cardiac rehabilitation approaches in minimizing triglycerides, anxiety, depression, waist circumference, and body mass index/weight of cardiac patients [18]. Two studies claimed the benefits of home-based cardiac rehabilitation in terms of reducing the social isolation levels of cardiac patients in the longer term [18,20]. One study affirmed the comparable efficacy of home/center-based cardiac rehabilitation measures in improving heart failure patients’ functional capacity and hemodynamic parameters [19]. One study advocated the potential of a home-based cardiac rehabilitation program to enhance patients’ self-efficacy with cardiovascular conditions [14]. Alternatively, only two studies confirmed the greater potential of center-based cardiac rehabilitation than home-based cardiac rehabilitation in improving the LVEF of cardiac patients [11,19]. One study affirmed the role of center-based cardiac rehabilitation in improving the sedentary lifestyle of patients with cardiovascular complications [16]. One study revealed the potential of hospital-based cardiac rehabilitation in improving the quality-of-life scores and recovery rate of cardiac patients with ischemic heart disease [13]. The overall findings exhibited higher efficacy of home-based cardiac rehabilitation measures in improving the long-term outcomes of cardiac patients. Table 1 presents the baseline characteristics of the included studies.

**Table 1:** Baseline characteristics of the included studies.

Author	Sample size	Study design	Intervention	Inferences
Haddadzadeh et al. (2011) [11]	42 subjects with post-episode coronary artery disease	Randomized single-blinded trial	The assessment of the potential of center/home-oriented exercise-based cardiac rehabilitation for elevating left ventricular ejection fraction in patients with a clinical history of coronary artery disease	The center/home-based exercise intervention substantially elevated left ventricular ejection fraction in treated patients and effectively improved their long-term prognosis of coronary artery disease (46.9 ± 5.9 to 61.5 ± 5.3). The exercise-based cardiac rehabilitation proved superior to standard cardiac care irrespective of its center-based or home-based administration
Avila et al. (2018) [12]	90 subjects with coronary artery disease	Randomized controlled (unblinded) trial	The evaluation of the potential of telemonitoring-oriented, home-based cardiac rehabilitation in improving the physical fitness of coronary artery disease patients	The home-based cardiac rehabilitation measure effectively enhanced the overall physical fitness of coronary artery disease patients and improved their health-related quality of life in the longer term. The measurement of physical fitness of coronary artery disease patients relied on their 30-second average oxygen uptake levels (P-interaction = 0.03; P = 0.04)
Bravo-Escobar, et al. (2017) [13]	28 subjects with a moderate cardiovascular predisposition and coronary artery disease	Randomized controlled trial	The assessment of home-based versus hospital-based (mixed surveillance) cardiac rehabilitation across patients with ischemic heart disease	The home-based and hospital-oriented cardiac rehabilitation programs effectively improved the quality-of-life scores and recovery rate of cardiac patients with ischemic heart disease (−4.314 [95% confidence intervals: 11.414-2.787; p = 0.206]) (10.93 [95% confidence interval: 17.251-3.334, p = 0.007])
Poortaghi et al. (2013) [14]	80 subjects with coronary artery disease	Randomized controlled trial	The assessment of the therapeutic potential of interdisciplinary home-based versus center-based cardiac rehabilitation	The study findings affirmed statistically significant improvements in the self-efficacy of cardiac patients after attending home-based cardiac rehabilitation program (36.59 ± 5.65)
Xu et al. (2016) [15]	52 subjects with acute myocardial infarction	Randomized controlled trial	The evaluation of home-based versus center-based cardiac rehabilitation program to track its efficacy in improving left ventricular ejection fraction, global circumferential strain, global area strain, global radial strain, and global longitudinal strain among patients with a clinical history of myocardial infarction	The home-based cardiac rehabilitation measure effectively improved segmental strains and left ventricular ejection fraction in the setting of acute myocardial infarction (p < 0.05)
Hoeve et al. (2018) [16]	731 subjects with acute coronary syndrome	Randomized controlled trial	The assessment of the effectiveness of telephone-based versus standard cardiac rehabilitation versus interactive physical activity counseling sessions for patients with acute coronary syndrome	The standard/center-based cardiac rehabilitation with interactive physical activity sessions predominantly impacted the sedentary lifestyle or moderate-to-vigorous-intensity physical activity patterns within a timeframe of 3-18 months (OR: 1.91, p = 0.033) (OR: 2.14, p = 0.054). The telephonic cardiac rehabilitation sessions failed to improve the physical activity and sedentary lifestyle of acute coronary syndrome patients
Kraal et al. (2017) [17]	90 subjects with low/moderate risk for cardiac diseases/manifestations	A prospective randomized controlled trial	The assessment of the long-term effectiveness of telemonitoring-supported, home-based training for cardiac patients	The telemonitoring-oriented, home-based cardiac rehabilitation measure effectively improved the health-related quality of life, physical activity, physical fitness, and satisfaction levels of patients with a clinical history of coronary artery bypass grafting, percutaneous coronary intervention/revascularization, and acute coronary syndrome (unstable angina/myocardial infarction) (p < 0.01)
Whittaker and Wade (2014) [18]	120 cardiac patients	Randomized controlled trial	The evaluation of the long-term benefits of telehealth-supported, home-based cardiac rehabilitation compared to hospital-based cardiac rehabilitation	The telemedicine oriented, home-based cardiac rehabilitation intervention effectively improved health outcomes based on a marked reduction in triglycerides, anxiety, depression, waist circumference, and body mass index/weight of the cardiac patients. The home-based cardiac rehabilitation also reduced the social isolation level of the cardiac patients. The long-term beneficial outcomes of home-based cardiac rehabilitation program surpassed the outcomes of hospital-based cardiac rehabilitation interventions
Karapolat et al. (2009) [19]	74 patients with a clinical history of heart failure	Randomized controlled trial	The assessment of the efficacy of hospital-based and home-based exercise programs in the setting of heart failure	The home-based and center-based cardiac rehabilitation program effectively improved the left ventricular ejection fraction, depression episodes, health-related quality of life, functional capacity, and hemodynamic parameters of heart failure patients. The home-based cardiac rehabilitation program provided significant therapeutic benefits compared to the center-based cardiac rehabilitation measures
Arthur et al. (2002) [20]	242 cardiac patients with coronary artery bypass graft	Randomized controlled trial	The assessment of therapeutic benefits of home-based versus hospital-based cardiac rehabilitation	The home-based exercise training (compared to hospital-based exercise training) effectively improved the health-related quality of life and social support of cardiac patients with coronary artery bypass graft status within the tenure of 3-6 months (51.2 ± 6.4 versus 48.6 ± 7.1 = 0.004) (36.0 ± 4.9 versus 34.6 ± 6.4 = 0.05)

Discussion

This systematic review affirmed the high therapeutic efficacy of home-based cardiac rehabilitation programs compared to center/facility-based cardiac rehabilitation interventions for patients with cardiovascular morbidities [11-20]. Home-based cardiac rehabilitation programs exhibit greater potential in enhancing the recovery and wellness of cardiac patients in the longer term. They also prove conducive to improving the prognostic outcomes of various cardiovascular conditions. Home-based cardiac rehabilitation measures exhibit the potential to effectively improve LVEF, HRQOL, physical fitness, recovery rate, self-efficacy, sedentary lifestyle, moderate-to-vigorous-intensity physical activity, satisfaction levels, and functional capacity of cardiac patients. They also prove beneficial in minimizing the levels of anxiety, depression, waist circumference, body mass index, and triglycerides in the setting of cardiovascular diseases. Home-based cardiovascular rehabilitation programs further help improve cardiac patients’ social support and hemodynamic parameters.

Our findings expanded the outcomes of the previously reported systematic review/meta-analyses by Imran et al. (2019) [7] and Anderson et al. (2017) [8], emphasizing the short-term effectiveness of center/home-based cardiac rehabilitation in improving functional capacity and HRQOL of cardiac patients. Our findings advocate the customization of home-based cardiac rehabilitation programs based on cardiac patients’ evolving preferences and needs to improve their prognosis, recovery rate, and satisfaction levels [21]. The expanded benefits of home-based cardiac rehabilitation measures also indicate their potential to reduce the risk of comorbid conditions in patients with cardiovascular diseases. For example, the potential of home-based cardiac rehabilitation programs to minimize triglyceride levels in cardiac patients indicates their efficacy in minimizing cardiovascular comorbidities. Our findings also advocate the positive implications of the telerehabilitation of cardiac patients. Telemedicine-oriented cardiac rehabilitation programs exhibit the capacity to improve the long-term health outcomes of remotely located patients with cardiovascular diseases [22]. Our findings further strengthen the requirement of viably replacing home-based and telehealth-supported cardiac rehabilitation programs for improving the long-term wellness outcomes of patients with cardiovascular diseases [23]. The reported results also strengthen the need to optimize home-based cardiac rehabilitation measures to improve the treatment benefits for cardiac patients.

The cardiac rehabilitation interventions reciprocate with multidisciplinary approaches that cater to patients with cardiovascular diseases’ personalized health care requirements [24]. These approaches assist in improving graded physical activity, health behaviors, glycemic control, lipid levels, and blood pressure of cardiac patients. Patients with a clinical history of stable angina, heart failure, stent placement, bypass surgery, and myocardial infarction particularly require cardiac rehabilitation to enhance their long-term medication adherence, functional capacity, and mental health. The cardiac rehabilitation measures also reduce the predisposition of cardiac patients to comorbid conditions. Smoking cessation counseling, psychological support, and nutritional counseling via cardiac rehabilitation programs help improve cardiac patients’ overall recovery capacity and treatment results [25]. The administration of home-based cardiac rehabilitation measures accordingly promises to enhance the long-term clinical outcomes of patients with cardiovascular complications.

The findings of our study were limited by its small sample size and a high risk of bias concerning allocation concealment and blinding of participants/outcome assessment. In addition, not all cardiovascular pathologies (heart attacks, bypass surgery, heart failure, valve diseases, etc.) were taken into account in the analysis. The lack of statistical analysis of the outcome variables further restricted the generalizability of our results across larger patient groups in the setting of cardiovascular diseases. Future studies should analyze and reinvestigate the long-term wellness enhancement potential of home-based cardiac rehabilitation programs for cardiac patients. They should also analyze the scope of replacing center-based cardiac rehabilitation measures with home-based interventions to improve long-term clinical outcomes and reduce patients’ overall healthcare burden with cardiovascular conditions.

## Conclusions

Home-based cardiac rehabilitation programs exhibit the potential to replace center-based cardiac rehabilitation measures in the context of improving long-term health and wellness outcomes of patients with cardiovascular diseases. The prolonged adherence of cardiac patients to home-based cardiac rehabilitation interventions may improve their cardiopulmonary fitness, mental health, satisfaction levels, and health-appropriate behaviors while minimizing their predisposition to comorbidities and adding to their quality-adjusted life years.
